# Quantitative proteomic analysis of *Pseudomonas pseudoalcaligenes* CECT5344 in response to industrial cyanide-containing wastewaters using Liquid Chromatography-Mass Spectrometry/Mass Spectrometry (LC-MS/MS)

**DOI:** 10.1371/journal.pone.0172908

**Published:** 2017-03-02

**Authors:** María Isabel Ibáñez, Purificación Cabello, Víctor Manuel Luque-Almagro, Lara P. Sáez, Alfonso Olaya, Verónica Sánchez de Medina, María Dolores Luque de Castro, Conrado Moreno-Vivián, María Dolores Roldán

**Affiliations:** 1 Departamento de Bioquímica y Biología Molecular, Edificio Severo Ochoa, Campus de Rabanales, Universidad de Córdoba, Córdoba, Spain; 2 Departamento de Botánica, Ecología y Fisiología Vegetal, Edificio Celestino Mutis, Campus de Rabanales, Universidad de Córdoba, Córdoba, Spain; 3 Departamento de Química Analítica, Edificio Marie Curie, Campus de Rabanales, Universidad de Córdoba, Córdoba, Spain; MJP Rohilkhand University, INDIA

## Abstract

Biological treatments to degrade cyanide are a powerful technology for cyanide removal from industrial wastewaters. It has been previously demonstrated that the alkaliphilic bacterium *Pseudomonas pseudoalcaligenes* CECT5344 is able to use free cyanide and several metal−cyanide complexes as the sole nitrogen source. In this work, the strain CECT5344 has been used for detoxification of the different chemical forms of cyanide that are present in alkaline wastewaters from the jewelry industry. This liquid residue also contains large concentrations of metals like iron, copper and zinc, making this wastewater even more toxic. To elucidate the molecular mechanisms involved in the bioremediation process, a quantitative proteomic analysis by LC-MS/MS has been carried out in *P*. *pseudoalcaligenes* CECT5344 cells grown with the jewelry residue as sole nitrogen source. Different proteins related to cyanide and cyanate assimilation, as well as other proteins involved in transport and resistance to metals were induced by the cyanide-containing jewelry residue. GntR-like regulatory proteins were also induced by this industrial residue and mutational analysis revealed that GntR-like regulatory proteins may play a role in the regulation of cyanide assimilation in *P*. *pseudoalcaligenes* CECT5344. The strain CECT5344 has been used in a batch reactor to remove at pH 9 the different forms of cyanide present in industrial wastewaters from the jewelry industry (0.3 g/L, *ca*. 12 mM total cyanide, including both free cyanide and metal−cyanide complexes). This is the first report describing the biological removal at alkaline pH of such as elevated concentration of cyanide present in a heterogeneous mixture from an industrial source.

## Introduction

Several billion liters of liquid wastes containing cyanides are produced at large scale by industrial activities such as mining and metal processing, electroplating, goal coking and nitrile polymers synthesis [1]. Mining and jewelry industries use cyanide in the processes for extracting gold, silver, copper and zinc from ores, and they are considered the largest producers of these highly toxic wastewaters. Cyanide-containing wastewaters often contain heavy metals and metalloids that increase their toxicity, making them hazardous effluents difficult to remove from the environment. In these wastewaters cyanide is often found as free ion (CN^−^) as well as metal−cyanide complexes [1]. The jewelry industry in the city of Córdoba (Spain) produces about 10 tons/year of an alkaline waste (pH >13) that contains approximately 40 g/L cyanide (*ca*. 1.5 M), including free cyanide (CN^−^ in dissolution) and both weak and strong metal–cyanide complexes [2,3,4]. These effluents must not be discharged without decreasing cyanide concentrations to levels lower than 1 mg/L. Physical and chemical treatments have been described for removing cyanide, although most of them are not currently applied [5,6,7]. Economic and environmental considerations make biological technologies useful in the removal of cyanide from industrial wastewaters, and several biological treatments have been described for detoxification of free cyanide and some metal–cyanide complexes present in mining wastewaters [7,8,9].

Cyanide toxicity is related with its capacity for binding to metal cofactors present in many metalloproteins, which become inhibited [1,10,11]. Thus, cyanide inhibits cytochrome *c* oxidase blocking aerobic respiration [12,13]. However, there are a variety of microorganisms able to deal with cyanide by displaying different degradation pathways, which lead to the formation of products like ammonium and CO_2_ [10,14,15]. Most microorganisms able to degrade cyanide operate at a neutral pH, where 99% cyanide is found as volatile HCN. Therefore, several considerations must be regarded for bioremediation of cyanide-rich effluents, including the requirement for an alkaliphilic microorganism (pH ≥ 9.0 with CN^−^/HCN in equilibrium or shifted to CN^−^).

The presence of metals and metal-cyanide complexes in industrial wastes requires the use of a microorganism able to tolerate metal toxicity and to degrade metal–cyanide complexes. Copper, iron and zinc, among other metals, are required at very low concentrations by microorganisms. However, metal accumulation at high concentrations cause cell toxicity because metals interact with cellular thiol components like glutathione [16]. Under aerobic conditions, copper can catalyze a Fenton-like reaction producing superoxide and other reactive oxygen species that may cause lipid peroxidation and protein damage [17]. The inherent capability of copper to cause damage has been addressed by bacteria with specific resistance mechanisms. Copper adopts two different redox states, the oxidized form Cu(II) and the reduced Cu(I) form, which is more reactive toward sulfhydryl groups. In the γ-proteobacterium *E*. *coli*, copper homeostasis includes a Cu(I)-translocating P-type ATPase (CopA) for export of copper from the cytoplasm to the periplasm, a membrane-bound cupric reductase with the active site facing the periplasm (Ndh-2), a periplasmic multicopper oxidase (CueO), and a tetra-component copper efflux pump (CusCFBA) that belongs to the RND (resistance-nodulation-cell division) proteins for extrusion of Cu(I) from the cytoplasm [17]. The β-proteobacterium *Cupriavidus* (*Ralstonia*) *metallidurans* CH34, which was isolated from a zinc decantation tank, is able to resist high concentrations of heavy metals. This strain contains about 40 systems involved in metal detoxification, including 10 P-type ATPases for zinc and copper extrusion, 20 RND-type proteins for zinc, copper, cadmium, cobalt, nickel and silver extrusion, and 3 cation diffusion facilitator (CDF) systems for cobalt, cadmium, nickel and iron extrusion [16]. In general, the systems for metal transport are not well characterized, and only in the case of iron different transport systems have been described. Many microorganisms secrete to the media small molecules called siderophores that bind iron. Fe^3+^-siderophores are incorporated by specialized transporters, which usually are ABC-type ATP-dependent or TonB-dependent systems [18]. Bacteria often regulate iron metabolism in response to iron availability and this regulation is mediated by the ferric-uptake regulator protein (Fur) that controls the iron-dependent expression of many genes [19].

*Pseudomonas pseudoalcaligenes* CECT5344 was isolated from the Guadalquivir River (Córdoba, Spain) by enrichment cultivation with cyanide [2,3]. This bacterium is able to use free cyanide, metal–cyanide complexes like nitroprusside, and other cyano-derivatives like cyanate or 2-hydroxynitriles (cyanohydrins) as the sole nitrogen source at alkaline pH [2,3,4,20]. The strain CECT5344 is able to grow in batch reactor with 2 mM sodium cyanide as the sole nitrogen source [21]. The complete genome sequence of *P*. *pseudoalcaligenes* CECT5344 has been elucidated [22,23,24], becoming a useful tool to develop transcriptomic and proteomic approaches to analyze bacterial responses to cyanide and metals. Recently, *P*. *pseudoalcaligenes* CECT5344 DNA microarrays have been developed to characterize the response to cyanides, including sodium cyanide and cyanide-containing wastewaters from the jewelry [25].

In this work, a quantitative proteomic approach by LC-MS/MS has been used to identify the mechanisms by which the strain CECT4344 deals with cyanide and metals during assimilation of cyanide present in a liquid residue from the jewelry industry. The cyanide removal from the jewelry industry wastewater, which contains high concentrations of both free cyanide and metal–cyanide complexes, has been achieved by using the bacterial strain *P*. *pseudoalcaligenes* CECT5344 in a batch reactor.

## Materials and methods

### Chemicals

The industrial cyanide-containing waste from the jewelry was supplied by Avenir S.L. and Gemasur S.L. (Córdoba, Spain). Ammonium chloride, sodium acetate, sodium malate, sodium citrate, and sodium octanoate were supplied by Sigma–Aldrich (St. Louis-MO, USA). Nitrogen was purchased from Air Liquid (Paris, France). All other chemicals used in the study were of analytical grade. Solutions were prepared by using Milli-Q water (Millipore, Bedford-MA, USA). Wastes containing cyanide or other toxic chemicals were handled and disposed by the Environmental Protection Unit, University of Córdoba.

### Bacterial growth determination

Bacterial growth was determined by following the absorbance at 600 nm (A_600_) in a spectrophotometer, by protein quantification [26] or by the estimation of the number of colony forming units (CFU), which was calculated from plate counts of diluted samples in LB-rich solid medium (plates were incubated at 30°C for 24 h and colonies of viable bacteria were counted), giving data as CFU/mL.

### Bacterial culture conditions

To determine the toxicity of copper, zinc and iron, *P*. *pseudoalcaligenes* CECT5344 cells were cultured in the phosphate-containing minimal medium M9 [27] supplemented with the corresponding metal at a concentration ranged from 0.5 to 15 mM in 125 mL Erlenmeyer flasks filled to 20% of their total volume with medium. The pH was adjusted to 9.5 with 0.1 N NaOH.

Experiments in bioreactor were conducted in a 10 L Biostat^®^ C plus reactor (Sartorius BBI systems, Melsungen, Germany), which was loaded with M9 mineral medium [27] containing 100 mM sodium acetate as carbon source. The medium was autoclaved, with exclusion of the MgSO_4_ and FeSO_4_ solutions, which were sterilized by filtration and added to the M9 trace solution after autoclaving. Initially, 4 mM ammonium chloride was added to the reactor as nitrogen source. After ammonium consumption, a 125-fold diluted wastewater containing 12 mM total cyanide (8 mM free cyanide and weak metal–cyanide complexes plus 4 mM strong metal–cyanide complexes) was added as the sole nitrogen source. The temperature was kept at 30°C and the pH, initially adjusted to 9.0, was controlled and kept constant at 9.0 by automatic addition of 1 N NaOH. Continuous agitation at 450 rpm was provided. The dissolved oxygen saturation was kept at 10% by means of pulse air control valves regulated by the main control unit in response to a sterile pO_2_ probe. In order to prevent HCN losses, the reactor exhaust cooler was connected to a washing flask with 0.5 N NaOH. The absence of cyanide in samples from this flask confirmed the effectiveness of the proposed procedure.

### Analytical determinations

Ammonium concentration in the culture media was determined as described previously [28]. Ammonium concentration in the jewelry residue was estimated by using the same method preparing a 125-fold dilution of the residue in M9 medium. Determination of cyanate concentration was based on its chemical conversion into ammonium in the presence of 0.1 ml of 6 M HCl and heat (1 min at 100°C). Cyanate concentration was calculated from the difference in the ammonium concentration before and after treatment [29]. Nitrite was determined colorimetrically as previously described [30]. Nitrate absence in the jewelry wastewater was confirmed by preparing a 125-fold dilution of this residue in M9 medium, which was incubated with zinc dust to chemically convert nitrate into nitrite.

The concentration of free and weak metal–cyanide complexes was determined colorimetrically [31]. Total cyanide (free, weak metal–cyanide complexes and stable cyanide–complexes) was determined in a manifold system [32], including a four-channel Gilson Minipuls-3 peristaltic pump and a photodissociation reactor with a reaction coil (1.25 × 0.8 mm internal diameter) around a 20 W UV lamp (Philips, Eindhoven, The Netherlands) coupled to a pervaporation cell [33]. A cyanide-selective electrode (model 6.0502.130, Metrohm, Hrisau, Switzerland) was fitted to the upper part of the pervaporation module facing the acceptor side of the gas-diffusion membrane. Samples were injected into a 0.3 M HNO_3_ solution stream and passed through the photodissociation reactor to break metal–cyanide complexes, obtaining HCN in the lower chamber of the pervaporation cell, which evaporates to the upper chamber of the cell trapping it into 0.1 M NaOH for cyanide determination by the cyanide-selective electrode [33]. Stock standard solutions (100 mg/mL) of CN^−^, Cu(CN)_4_^2–^, Fe(CN)_6_^3–^, Fe(CN)_6_^4–^, and Zn(CN)_4_^2–^ were prepared by dissolving the corresponding potassium salts in 0.01 M aqueous sodium hydroxide.

The metal content of the jewelry residue was determined by atomic absorption spectrometry. A spectrophotometer with deuterium background correction, Spectra 110, from Varian (Mulgrave, Victoria, Australia) was used. Copper, zinc and iron were determined by flame atomic absorption spectrophotometry (FAAS) with a 10 cm burner head and standard air–acetylene flame. Varian hollow cathode lamps were used as radiation sources in all instances. A shaker was used to assist the digestion step, and centrifugation step was applied to remove solid particles from the digestion of the sediment. Stock solutions (1 g/L final concentration) were prepared with CuSO_4_ (Panreac, Barcelona, Spain), and granular Fe or Zn (Sigma-Aldrich, St. Louis-MO, USA). Air and acetylene were used in FAAS as support and fuel gas, respectively. Each calibration plot was run with nine standard solutions within the linear calibration range and all measurements were the average of at least three replicates using the peak height as instrument signal.

### Proteomic analysis

*P*. *pseudoalcaligenes* CECT5344 cells were grown in two 250 mL conical flasks with M9 medium containing 100 mM sodium acetate and 2 mM ammonium chloride. After this nitrogen source was depleted (approximately 24 h), the jewelry wastewater (containing 2 mM cyanide) was added to one of the cultures, whereas 2 mM ammonium chloride was added to a second conical flask as alternative nitrogen source. To carry on with the quantitative LC-MS/MS four biological replicates were set up for each nitrogen source. Cultures were monitored until 50% of the total nitrogen source (cyanide or ammonium) was consumed, and then cells were harvested (at the middle of the log-phase) by centrifugation at 12000 × *g* for 15 min and kept at -80°C until use. Samples for LC-MS/MS proteomic analysis were prepared by resuspension of frozen cells in Tris-HCl buffer (50 mM, pH 8) containing 4% CHAPS and 8 M urea, then cells were broken by cavitation as previously described [34]. Proteins were cleaned with the 2-D Clean-UP Kit (GE Healthcare, Little Chalfont, UK) and after precipitation were resuspended in a solution containing 6 M urea. Protein concentration was estimated by the Bradford method [35] and sample concentration ranged from 2 to 4 μg/μL. Samples were digested with trypsin overnight at 37°C with top-down agitation. All analyses were performed with a Dionex Ultimate 3000 nano UHPLC system (Thermo Fisher Scientific, Waltham-MA, USA) connected to a mass spectrometer LTQ Orbitrap XL (Thermo Fisher Scientific, Waltham-MA, USA) equipped with nanoelectrospray ionization interface. The separation column was Acclaim Pepmap C18, 150 mm × 0.075 mm, 3 μm pore size (Thermo Fisher Scientific, Waltham-MA, USA). For trapping of the digest, it was used a 5 mm × 0.3 mm precolumn Acclaim Pepmap C18 (Agilent Technologies, Waldbronn, Germany). One fourth of the total sample volume, corresponding to 5 μL, was trapped at 10 μL/min flow rate, for 5 min, with 2% acetonitrile/0.05% trifluoroacetic acid. After that, the trapping column was switched on-line with the separation column and the gradient was started. Peptides were eluted with a 60-min gradient of 5–40% acetonitrile/0.1% formic acid solution at a 300 nL/min flow rate. MS data (Full Scan) were acquired in the positive ion mode over the 400–1500 m/z range. MS/MS data were acquired in dependent scan mode, selecting automatically the five most intense ions for fragmentation, with dynamic exclusion set to on. In all cases, a nESI spray voltage of 1.9 kV was used. Tandem mass spectra were extracted using Thermo Proteome Discoverer 1.4 (Thermo Fisher Scientific, Waltham-MA, USA). Charge state deconvolution and deisotoping were not performed. The raw data was processed using Proteome Discoverer (version 1.4, Thermo Scientific). MS2 spectra were searched with SEQUEST engine against a database of *P*. *pseudoalcaligenes* CECT5344 (deposited in the EMBL database under the accession number HG916826). Peptides were generated from a tryptic digestion with up to one missed cleavages, carbamidomethylation of cysteines as fixed modifications, and oxidation of methionine as variable modifications. Precursor mass tolerance was 10 ppm and product ions were searched at 0.8 Da tolerances. Peptide spectral matches (PSM) were validated using percolator based on q-values at 1% FDR (False Discovery Rate), calculated against concatenated decoy database. With Proteome Discoverer, peptide identifications were grouped into proteins according to the law of parsimony and filtered to 1% FDR. For proteins identified from only one peptide, fragmentations were checked manually.

The bioinformatic analysis of protein sequences included computational predictions of subcellular localization that were carried out using the PSORTb program version 3.0.2. (http://www.psort.org/psortb/index.html) and predicted protein functions were retrieved from the UniProt knowledge base (http://www.uniprot.org). Proteins shared in both nitrogenous sources, the cyanide-containing jewelry wastewater and ammonium chloride, were quantified by using the Progenesis IQ software considering a *p*-value (Anova) ≤ 0.05 and a fold change ≥ 2. In the case of proteins exclusively induced by the cyanide-containing jewelry wastewater (not found in ammonium), all peptides detected in the four biological samples were considered when these four replicates displayed a fold change < 2. Proteins were quantified by using the XIC (Extracted-Ion Chromatogram) on the three most intense peptides. The mass spectrometry proteomics data have been deposited to the ProteomeXchange Consortium (http://proteomecentral.proteomexchange.org) via the PRIDE partner repository [36] with the dataset identifier PXD005056.

### Mutagenesis of the BN5_1894 gene encoding a GntR-like protein

A mutant strain of *P*. *pseudoalcaligenes* CECT5344 was generated by insertion of a kanamycin cassette in a central region of the BN5_1894 gene that codes for a GntR-like bacterial transcription factor. Genomic DNA was isolated as indicated by the manufacturers (Wizard Genomic Purification Kit, Promega). The *gntR* BN5_1894 gene was amplified from genomic DNA by PCR with the oligonucleotides: gntR-F: 5′- AAGAATTCTACCAGCAGATAGCCCGCCAGAT-3′ (*Eco*RI site is underlined) and gntR-R: 5′-AAGCATCCGTCAGGTTCTCCGCGGCAATCAC-3′ (*Bam*HI site is underlined) to yield a 512 bp DNA fragment. The PCR fragment was cloned into pGEMT-easy vector and subsequently subcloned into the pK18mob suicide vector with the restriction sites *Eco*RI and *Bam*HIII. This mobilizable construct was transferred to the wild-type strain by conjugation and transconjugants were selected by homologous recombination in media with nalidixic acid and kanamycin.

### Statistics

All plotted data in each figure represent the averages of three independent experiments. The mean standard deviations never exceeded 5%.

## Results

### Analytical composition of the jewelry residue

Wastewaters from the jewelry industry usually contain large amounts of cyanide and display an extremely alkaline pH (>13). The concentration of metals in the jewelry wastewater from the city of Córdoba (Spain) was investigated by applying atomic absorption spectrometry (Table 1). Copper was the most abundant metal in this liquid residue (285 mM), followed by zinc (147 mM) and iron as the less abundant (35 mM), but this method is unable to distinguish free and cyanide–complexed metals. To detect total cyanide present as free ion and weak–or strong metal–cyanide complexes in the jewelry wastewater the pervaporation method has been used, and a concentration of 39 g/L (*ca*. 1.5 M) total cyanide was established. The alternative method based on a colorimetric determination of cyanide revealed that in this wastewater 26 g/L cyanide (*ca*. 1 M) was present as free (ion) cyanide or weak metal–cyanide complexes, mainly with copper as the most abundant metal. However, this colorimetric method does not detect strong metal–cyanide complexes and, therefore, the remainder of total cyanide concentration, 13 g/L cyanide (*ca*. 0.5 M), is probably cyanide strongly bound to metals, such as iron-cyanide complexes. Very small concentrations of other nitrogenous compounds were also identified, including 3.6 mg/L ammonium (*ca*. 200 μM), 3.8 mg/L cyanate (*ca*. 90 μM) and 0.37 mg/L nitrite (*ca*. 8 μM).

**Table 1:** Determination of metals in the jewelry residue by flame atomic absorption spectrometry.

Parameter	Cu	Fe	Zn
λ (nm)	324.7	248.3	231.9
*A (mU)	4	5	5
^+^SW (nm)	0.5	0.2	1
Background correction	No	Yes	Yes
Working range (μg/mL)	0.05–0.4	0.05–0.4	0.05–0.4
Metal concentrations (mM)	285±6	34.7±2	147.5±5

*A: Absorbance.

^+^SW: Slit width.

### Proteomic analysis of *P*. *pseudoalcaligenes* CECT5344 cells grown with the jewelry wastewater

**Fig 1:**
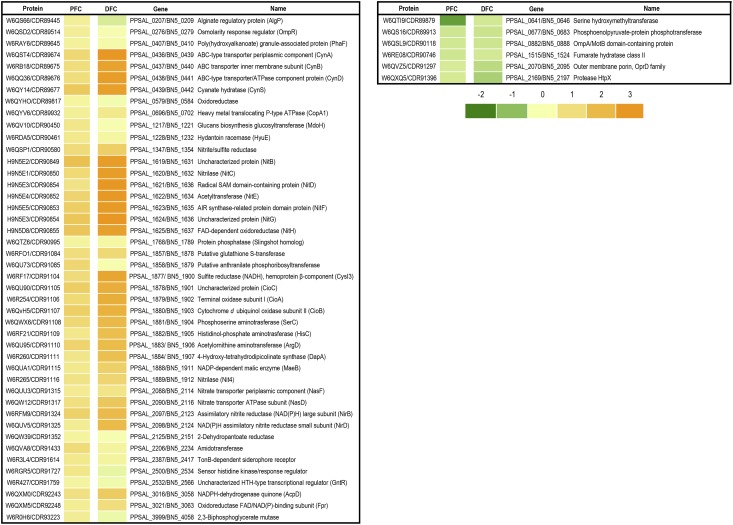
Heat-map of *P*. *pseudoalcaligenes* CECT5344 proteins detected by LC-MS/MS in a differential analysis from cells grown with the jewelry residue *versus* ammonium. Proteins induced or repressed in response to the jewelry residue are compared against genes induced or repressed by this cyanide-containing wastewater described in a previous transcriptomic analysis [25]. Only the induced proteins displaying the highest score values are shown in this figure. Score, fold change and *p*-value of all proteins are available in the PRIDE database (dataset identifier PXD005056). A log_10_ scale (from -2 to 3) has been used, with yellow-orange color for induction and green color for repression. Proteins were quantified by using the XIC (Extracted-Ion Chromatogram) on the three most intense peptides. FPC, protein fold change, DFC, transcript fold change. Protein and gene references correspond to UniProt and HG916826 accession numbers [23] and GenBanK and LK391695 accession numbers [24].

**Fig 2:**
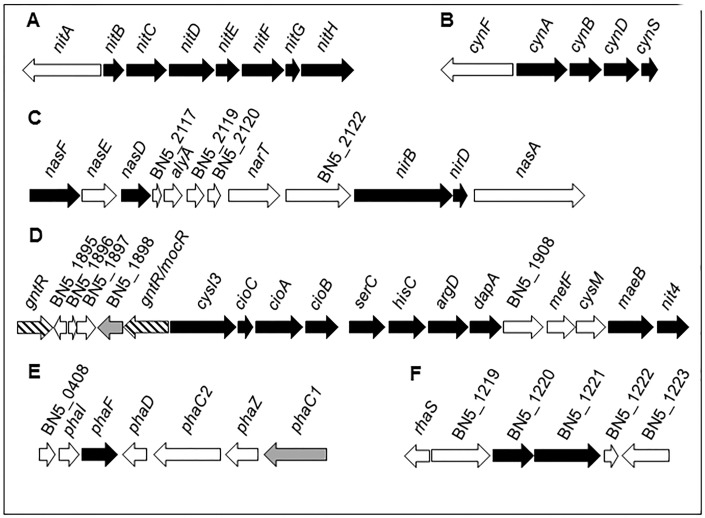
*P*. *pseudoalcaligenes* CECT5344 gene clusters containing genes that code for some proteins induced by the jewelry residue. The gene clusters include genes encoding some of the proteins induced by the jewelry residue (black arrows) described in Fig 1. Genes in the same cluster are drawn to scale and the number/name of each gene corresponds to the accession number HG916826 [23]. Other genes located in these clusters (white arrows) are the following: **A.** Sigma54-dependent transcriptional regulator (*nitA*, BN5_1630); **B.** Fis family transcriptional regulator (*cynF*, BN5_0438); **C.** Nitrate ABC transporter, inner membrane subunit (*nasE*, BN5_2115), hypothetical protein (BN5_2117), putative lyase (*alyA*, BN5_2118), putative thioesterase (BN5_2119), putative secreted protein (BN5_2120), nitrate transporter (*narT*, BN5_2121), protein kinase (BN5_2122), nitrate reductase (*nasA3*, BN5_2125); **D.** GntR family regulatory protein (BN5_1894), hypothetical protein (BN5_1895), hypothetical protein (BN5_1896), thiol-disulfide isomerase and thioredoxins protein (BN5_1897), 2-dehydro-3-deoxyphosphogluconate aldolase/4-hydroxy-2-oxoglutarate aldolase (BN5_1898), GntR family transcriptional regulator (*mocR*, BN5_1899), high-affinity glucose transporter (BN5_1908), methylenetetrahydrofolate reductase (*metF*, BN5_1909), cysteine synthase B (*cysM*, BN5_1910); **E.** Putative polyhydroxyalkanoic acid system protein (BN5_0408), polyhydroxyalkanoate granule-associated protein phasin (*phaI*, BN5_0409), TetR family transcriptional regulator (*phaD*, BN5_0411), polyhydroxyalkanoate synthase class II (*phaC2*, BN5_0412), polyhydroxyalkanoate depolymerase (*phaZ*, BN5_0413), polyhydroxyalkanoate polymerase (*phaC1*, BN5_0414); **F.** HTH-type transcriptional activator RhaS (BN5_1218), BglX periplasmic β-glucosidase (BN5_1219), CybB3 cytochrome *b561* homolog (BN5_1222), UvrD/Rep helicase (BN5_1223). Two genes encoding GntR-like transcriptional regulators (see S3 Fig) are striped and genes encoding proteins induced specifically in response to the jewelry residue (see Table 2) are highlighted in grey.

A quantitative proteomic analysis by LC-MS/MS has been applied to cells grown with the jewelry wastewater as the sole nitrogen source and this growth condition has been compared to ammonium chloride grown cells, as described in Materials and Methods section. More than 150 proteins were induced by the jewelry wastewater when compared with ammonium as alternative nitrogen source, considering a fold change ≥ 2 and *p*-value ≤ 0.05 as significant parameters (dataset identifier PXD005056). From these induced proteins, those displaying the highest score values, and therefore probably playing a putative role in detoxification/assimilation of the cyanide-containing wastewater, are included in Fig 1. Previous to this work, a transcriptomic analysis in response to the jewelry residue of the strain CECT5344 has been performed [25]. To achieve a full range, some of the genes/proteins repressed by jewelry residue are also included in Fig 1. The levels of relevant proteins required for cyanide degradation like those involved in cyanide assimilation (NitB-H proteins) and cyanide-insensitive respiration (CioABC oxidase) are well correlated to the expression of their respective genes. Fig 2 shows several gene clusters coding for proteins induced by the residue, such as *nit*, *cyn* and *nas-nir* genes, which are involved in the assimilation of the nitrogenous sources cyanide, cyanate and nitrate/nitrite, respectively (Fig 2A–2C). Genes coding for proteins induced by the residue like the sulfite reductase CysI3, the cyanide-insensitive terminal oxidase CioABC, the malic enzyme MaeB and the 3-cyanoalanine nitrilase Nit4, among others, were also clustered together. Two *gntR*-like genes are also present in this gene cluster (Fig 2D). The PhaF polyhydroxyalkanoate granule-associated protein encoded by BN5_0410 and two proteins involved in glucans biosynthesis (BN5_0120 and BN5_0121 gene products) were also induced by the jewelry wastewater (Fig 2E and 2F). Additionally, the jewelry residue also induced the nitrite/sulfite reductase CysI1 encoded by a gene located close to the malate:quinone oxidoreductase *mqoB*, the TonB-dependent siderophore receptor encoded by BN5_2417 and several proteins related to metal resistance like the P-type ATPase metal extrusion protein CopA1 and the BN5_2091 gene product, which belongs to a RND-HME type metal extrusion system. Different putative regulatory proteins were also induced, such as the transcriptional activator RhaS encoded by BN5_4423 gene that is adjacent to putative genes coding for OxyR-type hydrogen peroxide-inducible activators (BN5_4224 and BN5_4225 genes), the flavoprotein Fpr (BN5-3063 gene product), and the sensor-histidine kinase response regulator encoded by the BN5-2534 gene that shares locus with other genes encoding an organic hydroperoxyde resistance transcriptional regulator (BN5-2535) and the glutathione peroxidase BsaA (BN5_2536). Also induced by the jewelry residue were the GntR regulatory protein encoded by BN5_2566 and an oxidoreductase encoded by the gene BN5_0584 that is located near the *gntR*-like regulatory genes BN5_0586 and BN5_0594. The functional analysis of the proteins induced by the jewelry residue (S1 Fig) revealed that most of them belong to the categories nitrogen compound metabolic processes (17 proteins), transcription/DNA-templated (3 proteins), nitrate assimilation (2 proteins), and glucan biosynthesis process (2 proteins), with only one protein included in each other categories.

**Fig 3:**
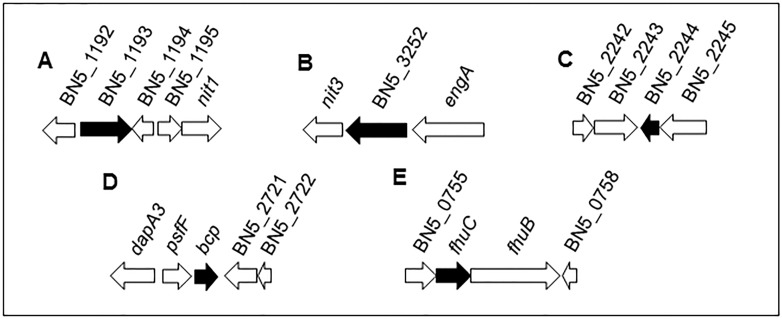
*Pseudomonas pseudoalcaligenes* CECT5344 gene clusters containing genes coding for proteins specifically induced by the jewelry residue. Five gene clusters (A-E) that include genes encoding proteins that are only found in cells grown with the jewelry residue (black arrows) are shown. Information about these proteins is presented in Table 2. Genes in the same cluster are drawn to scale and the number/name of each gene corresponds to the accession number HG916826 [23]. Other genes located in these clusters (white arrows) are the following: **A.** Hypothetical protein (BN5_1192), hypothetical protein (BN5_1194), hypothetical protein (BN5_1195), nitrilase homolog 1 (*nit1*, BN5_1196); **B.** Nitrilase/cyanide hydratase (*nit3*, BN5_3251), GTP-binding protein (*engA*, BN5_3253); **C.** Rhodanase domain-containing protein (BN5_2242), putative cytochrome *c*-related protein (BN5_2243), hypothetical protein (BN5_2245); **D.** Dihydrodipicolinate synthase (*dapA3*, BN5_2718), glycine cleavage system transcriptional repressor (*psfF*, BN5_2719), putative permease PerM (BN5_2721), permease PerM, C-terminal (BN5_2722); **E.** Hypothetical protein (BN5_0755), ferrichrome transport system permease protein FhuB (BN5_0757), hypothetical protein (BN5_0758).

A non-quantitative analysis carried out with the Progenesis IQ software has revealed that another 47 proteins were specifically induced by the jewelry residue (dataset identifier PXD005056) because they were undetectable in cells grown with ammonium (Table 2). Some of these proteins are encoded by genes belonging to clusters that include nitrilases, rhodanase, oxidative stress response or iron transport genes (Fig 3). Other induced proteins were involved in iron-sulfur centers assembly/maturation, metal and multidrug resistance, amino acid transport and metabolism or purine biosynthesis. The functional analysis of these proteins specifically induced by the jewelry residue (S2 Fig) revealed that most of them belong to the categories biosynthetic process (8 proteins), nitrogen compound metabolic process (6 proteins), transcription/DNA-templated (3 proteins) and regulation of RNA metabolic process (2 proteins), with only one protein included in each other categories.

**Table 2:** *P*.* pseudoalcaligenes* CECT5344 proteins exclusively found in the jewelry residue (detected by LC-MS/MS).

Accession number protein/gene^a^	Accession number protein/gene^b^	Protein name or function	Score	N^c^	FD^d^
CDR92386/PPSAL_3159	W6QY63/BN5_3204	Formamidase (AmiF)	8.10	3(3)	1.58
CDR89843/PPSAL_0605	W6QRU1/BN5_0610	Uncharacterized protein	6.85	4(4)	1.40
CDR92443/PPSAL_3216	W6R0Q3/BN5_3262	Uncharacterized protein (IscX)	4.87	2(2)	1.20
CDR89565/PPSAL_0327	W6RAR6/BN5_0330	Dihydropyrimidinase (HydA)	4.23	2(2)	1.46
CDR89934/PPSAL_0698	W6QTN1/BN5_0704	Heavy metal transport/detoxification protein (CopZ)	3.96	2(2)	1.61
CDR92106/PPSAL_2879	W6QZP0/BN5_2918	Putative tricarboxylic transport membrane protein (TctC)	3.45	2(2)	1.38
CDR93115/PPSAL_3891	W6R194/BN5_3950	Uncharacterized protein	2.77	2(2)	1.58
CDR91102/PPSAL_1875	W6QVH1/BN5_1898	2-dehydro-3-deoxy-phosphogluconate aldolase/4-hydroxy-2-oxoglutarate aldolase	2.71	1(1)	1.74
CDR90860/PPSAL_1630	W6QW58/BN5_1642	YecA family protein	2.51	2(2)	1.27
CDR92433/PPSAL_3206	W6R0P4/BN5_3252	Putative aminotransferase	2.50	2(2)	1.55
CDR93232/PPSAL_4008	W6R0I5/BN5_4068	Imidazole glycerol phosphate synthase subunit (HisH)	2.18	1(1)	1.45
CDR91982/PPSAL_2755	W6RHP8/BN5_2794	Uncharacterized protein	1.99	3(3)	1.30
CDR93365/PPSAL_4141	W6R3H6/BN5_4204	Heme-binding protein A	1.76	1(1)	1.81
CDR91911/PPSAL_2684	W6QZ60/BN5_2720	Alkyl hydroperoxide reductase (Bcp)	1.65	1(1)	1.39
CDR93072/PPSAL_3848	W6RKS4/BN5_3907	Diguanylate cyclase	1.64	2(2)	1.80
CDR90235/PPSAL_1002	W6QSY5/BN5_1005	Carboxylesterase	1.64	1(1)	1.42
CDR91506/PPSAL_2279	W6QY08/BN5_2307	AraC-family regulatory protein	1.54	1(1)	1.15
CDR92773/PPSAL_3546	W6QZ64/BN5_3598	GCN5-related N-acetyltransferase	1.44	1(1)	1.37
CDR93239/PPSAL_4015	W6R1K6/BN5_4076	Uncharacterized adenine-specific methylase (YeeA)	1.43	1(1)	1.86
CDR89403/PPSAL_0165	W6QXD6/BN5_0167	XRE family transcriptional regulator	1.36	1(1)	1.55
CDR91333/PPSAL_2106	W6QXH9/BN5_2132	4-hydroxy-4-methyl-2-oxoglutarate aldolase (oxaloacetate decarboxylase) (MenG)	1.17	1(1)	1.57
CDR92929/PPSAL_3705	W6QZR6/BN5_3762	Polyamine-transporting ATPase (PotA)	1.15	2(2)	1.33
CDR92785/PPSAL_3558	W6R0C0/BN5_3610	Uncharacterized protein	1.12	1(1)	1.59
CDR91455/PPSAL_2228	W6QWF9/BN5_2256	Putative two-component response regulator	1.12	1(1)	1.62
CDR89776/PPSAL_0538	W6QQC6/BN5_0543	Transglutaminase domain-containing protein	1.09	1(1)	1.51
CDR90756/PPSAL_1525	W6RE17/BN5_1534	4-hydroxyphenylacetate isomerase/decarboxylase (MhpD)	1.09	1(1)	1.51
CDR91985/PPSAL_2758	W6QY10/BN5_2797	Uncharacterized protein	1.08	1(1)	1.29
CDR89986/PPSAL_0750	W6QR17/BN5_0756	ATP-binding component of hydroxamate-dependent iron transport (FhuC)	1.08	1(1)	1.98
CDR91345/PPSAL_2118	W6QUY1/BN5_2144	Putative transcriptional regulator	1.06	2(2)	1.55
CDR91141/PPSAL_1914	W6R295/BN5_1937	Putative secreted protein	1.05	1(1)	1.88
CDR90298/PPSAL_1065	W6QZR7/BN5_1069	Uncharacterized protein	1.01	1(1)	1.61
CDR91549/PPSAL_2322	W6QWP5/BN5_2353	Uncharacterized protein	1.01	1(1)	1.86
CDR93568/PPSAL_4347	W6R1K1/BN5_4411	Heavy metal sensor histidine kinase (CusS)	0.96	1(1)	1.48
CDR92051/PPSAL_2824	W6QZJ0/BN5_2863	Histidine triad (HIT) protein	0.94	1(1)	1.47
CDR91443/PPSAL_2216	W6QVB9/BN5_2244	Transglutaminase domain-containing protein	0.85	2(2)	1.67
CDR89649/PPSAL_0411	W6QSQ8/BN5_0414	Poly(3-hydroxyalkanoate) polymerase (PhaC1)	0.70	1(1)	1.44
CDR90659/PPSAL_1428	W6QU70/BN5_1433	Uncharacterized protein	0.70	1(1)	1.34
CDR89436/PPSAL_0198	W6RAG3/BN5_0200	Sensory transduction protein kinase (AlgZ)	0.70	2(2)	1.85
CDR89332/PPSAL_0094	W6QP33/BN5_0094	Cytochrome c oxidase assembly protein (CtaG/Cox11)	0.57	1(1)	1.99
CDR93503/PPSAL_4282	W6R1E6/BN5_4346	Cobalamin synthetase W domain-containing protein 1 (P47K)	0.53	1(1)	1.85
CDR91516/PPSAL_2289	W6RG87/BN5_2318	Uncharacterized protein	0.44	1(1)	1.24
CDR92526/PPSAL_3299	W6R697/BN5_3346	Ferrochelatase-heme synthase (HemH)	0.37	1(1)	1.51
CDR90513/PPSAL_1280	W6R0E4/BN5_1285	HTH-type transcriptional activator ampR (GcvA)	0.36	1(1)	1.51
CDR91038/PPSAL_1811	W6QWP3/BN5_1832	Uncharacterized protein	0.27	1(1)	1.70
CDR90760/PPSAL_1529	W6QVX0/BN5_1538	Putative ring-cleaving dioxygenase	0.26	1(1)	1.34
CDR91500/PPSAL_2273	W6QWK0/BN5_2301	Furoyl-CoA synthetase (HmfD)	0.20	1(1)	1.13
CDR90422/PPSAL_1189	W6QS82/BN5_1193	Uncharacterized protein	0.20	1(1)	1.51

^(a)^ Protein and gene references from GenBanK and LK391695 accession numbers (Wibberg et al., 2016);

^(b)^ Protein and gene references from UniProt and HG916826 accession numbers (Wibberg et al., 2014);

^(c)^ N: peptide count, unique peptides are shown in parenthesis;

^(d)^ FD: a fold change < 2 has been considered for result significance (variation among biological replicates).

**Fig 4:**
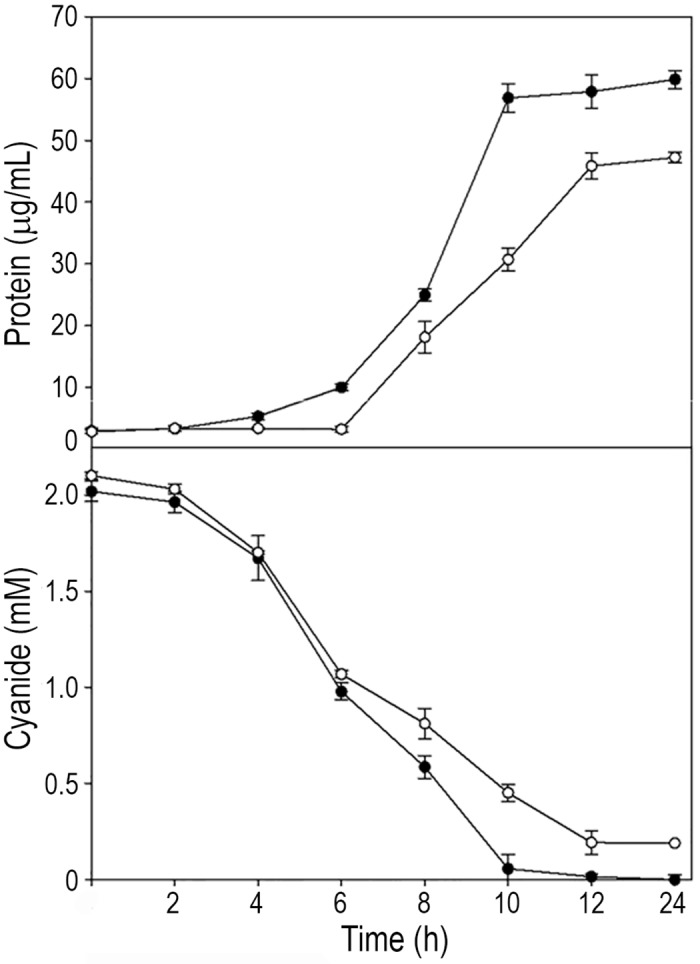
Growth and cyanide consumption of *P*. *pseudoalcaligenes* CECT5344 wild-type and GntR^−^mutant strains with the jewelry residue as the sole nitrogen source. Wild-type (filled symbols) and GntR^−^(empty symbols) strains were cultured in minimal media M9 with acetate as carbon source and the cyanide-containing jewelry residue as the nitrogen source. Bacterial growth (triangles) was estimated by protein concentration determination with a modified method of the Lowry procedure [26]. Total cyanide (circles) was determined as indicated in Material and Methods section.

A large number of genes coding for putative GntR-like regulatory proteins are present in the *P*. *pseudoalcaligenes* CECT534 genome [22,23,24]. The phylogenic tree of GntR regulatory proteins is represented (S3 Fig). The BN5_1894 gene is clustered together another *gntR*-like gene (*gntR*/*mocR*, BN5_1899) and both genes were found induced in the transcriptomic analysis carried out in response to the cyanide-containing liquid residue [25]. A mutational analysis of the *P*. *pseudoalcaligenes* CECT5344 *gntR*-like gene BN5_1894 has been carried out by insertion of a kanamycin cassette. When the jewelry residue was used as the sole nitrogen source, both growth and cyanide consumption were delayed in this GntR^−^mutant strain when compared to the wild-type (Fig 4). However, both strains showed similar growth when ammonium was used as nitrogen source (not shown).

### Batch reactor biodegradation of cyanide compounds from the jewelry industry

Basic parameters such as pH and oxygen concentration were initially optimized at 10% oxygen concentration and pH 9.0, which were kept constant during the cyanide degradation process in all experiments carried out in batch reactor. In order to minimize the cost of the process, additional parameters such as the carbon source, addition citrate as metal chelator and initial biomass have been considered to increase cyanide degradation rate, minimizing the operational time. The wastewater residue was added at a final 125-fold dilution to obtain a high concentration of total cyanide in the media (12 mM) and metal concentrations of about 2.3 mM copper, 0.3 mM iron and 1.2 mM zinc. The ability of *P*. *pseudoalcaligenes* CECT5344 to tolerate heavy metals was previously determined, and the strain CECT5344 was able to grow well in the presence of copper, zinc and iron (2.5 mM each), a metal concentration higher than those present in the culture media prepared after 125-fold dilution of the jewelry residue.

**Fig 5:**
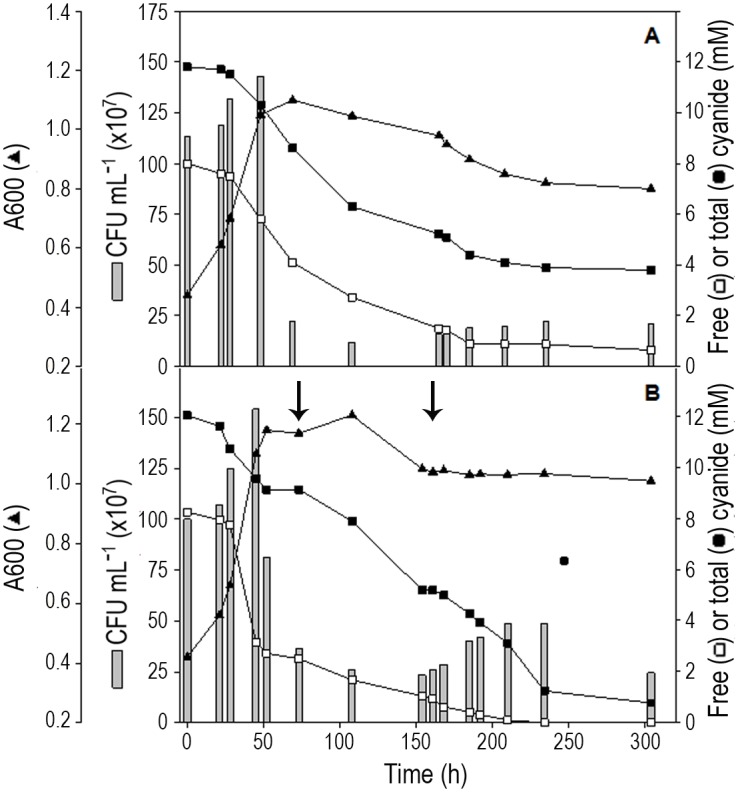
Bioremediation of cyanide from jewelry wastewaters. Cells were cultured at constant pH 9.0 in a 10 L reactor with 100 mM sodium acetate and 4 mM ammonium chloride. After ammonium depletion (time 0 in the figure), the jewelry residue was added to reach 0.3 g/L total cyanide concentration (*ca*. 12 mM) and bacterial growth was determined by estimating absorbance at 600 mn (▲) and CFU/mL (grey bars). Free cyanide plus weak metal–cyanide complexes (□) and total cyanide (■) concentrations were determined as indicated in Material and Methods section. A) Acetate as carbon source without citrate additions. B) Acetate as carbon source with additions of 17 mM citrate made at the times indicated by the arrows.

Acetate was the most suitable carbon source used by *P*. *pseudoalcaligenes* CECT5344 to deal with the removal of cyanide from the jewelry residue at constant pH 9.0 in the batch reactor. Under these conditions (12 mM total cyanide), free cyanide was successfully consumed but some metal–cyanide complexes remained in the media with acetate because 3.8 mM total cyanide was still present in the media after 300 h growth (Fig 5A). Additions of 17 mM citrate as a metal chelator not only caused the complete removal of free cyanide, but also a remarkable increase of total cyanide degradation, because the metal–cyanide complexes were almost completely removed from the medium, with only 5% total cyanide (0.65 mM) remaining at the end of the experiment (Fig 5B). A total cyanide consumption rate of about 1.9 mg cyanide/L×A_600_×h was achieved under these experimental conditions.

## Discussion

Wastewaters containing high concentrations of cyanide are produced worldwide as a consequence of different industrial activities [6,7]. In addition, metals are usually present in the residues from mining and jewelry industries, making them even more poisonous. In this work, the metal composition of the jewelry wastewater has been shown to contain iron, zinc and copper at concentrations ranging from 35 to 285 mM (Table 1). Simple and easy methods to determine colorimetrically free cyanide and weak metal–cyanide complexes have been described [31]. However, analytical determination of the most stable metal–cyanide complexes remains complicated. Weak metal–cyanide complexes include zinc, nickel, copper and cadmium, whereas the more stable metal–cyanide complexes involve cobalt and iron [37]. The volatile nature of cyanide has propitiated its automated determination after gas diffusion using flow injection manifolds [38,39,40]. Nevertheless, the use of pervaporation for separation of volatile analytes constitutes an attractive alternative over gas diffusion because it avoids clogging or deterioration of the membrane [41]. The method successfully established in this work for determination of total cyanide is based on UV-photodissociation of weak metal–cyanide complexes and strongly metal–cyanide complexes, followed by pervaporation of the target analyte and potentiometric determination by a cyanide-selective electrode after collection by a diluted NaOH solution.

The strain *P*. *pseudoalcaligenes* CECT5344 is able to grow with free cyanide and some metal−cyanide complexes like ferric−, ferrous− and copper−cyanide complexes as the sole nitrogen source [2,3]. To understand the complex processes involved in cyanide and metal resistance and detoxification, we have carried out a quantitative proteomic analysis by LC-MS/MS in cells grown with the jewelry residue compared to ammonium grown cells (Fig 1). The functional categories established accordingly to the GO analysis for the proteins induced by the jewelry residue found in the proteomic analysis are diverse, but mainly related to nitrogen compound metabolism, transport and biosynthetic processes (S1 Fig).

The analysis of the complete genome sequence of the strain CECT5344 has revealed the existence of a wide variety of gene clusters involved in the assimilation of different nitrogenous sources [22,23,24]. The jewelry wastewater is a complex mixture of different chemical compounds, and in addition to large amounts of cyanides, other nitrogenous compounds like cyanate, ammonium and nitrite are also present in the residue, although at very low concentrations (< 300 μM) that cannot support bacterial growth. However, this could explain that in the proteomic analysis of the strain CECT5344, the cyanate transporter components CynABD, the cyanase CynS, and several proteins required for nitrate/nitrite assimilation were induced in response to the jewelry residue (Figs 1, 2B and 2C). As *P*. *pseudoalcaligenes* CECT5344 harbors the *cynFABDS* gene cluster for cyanate assimilation [29], this strain can be used for removal of industrial residues that often contain both cyanide and cyanate as co-contaminants. Two nitrilases, NitC and Nit4, have been found induced by the jewelry residue (Figs 1, 2A and 2D). The nitrilase NitC is essential for cyanide assimilation in *P*. *pseudoalcaligenes* CECT5344. The cyanide degradation pathway in this bacterial strain includes a malate:quinone oxidoreductase that converts malate into oxaloacetate, which reacts chemically with cyanide to form a nitrile, and the nitrilase NitC hydrolizes this compound to generate ammonium, thus enabling this bacterium to grow with cyanide as the sole source of nitrogen [20,42]. All structural proteins NitB-H encoded by *nit1C* gene cluster were also found induced by the jewelry residue (Figs 1 and 2A). A preliminary analysis by 2D-PAGE analysis in *P*. *pseudoalcaligenes* CECT5344 revealed the induction by sodium cyanide of only a few proteins, including NitB and NitG proteins, thus confirming the essential role of the *nit1C* gene cluster in cyanide assimilation [20,42]. The nitrilase Nit4 is of unknown function, although displays homology with 3-cyanoalanine nitrilases. The role of 3-cyanoalanine or some amino acids in cyanide detoxification or assimilation has not been yet elucidated. Two other nitrilase genes, *nit1* and *nit3*, are also present in the *P*. *pseudoalcaligenes* CECT5344 genome, and both of them are clustered together genes coding for proteins that were specifically induced by the jewelry residue (Fig 3A and 3B).

The jewelry residue contains copper, iron and zinc (Table 1), and in the genome of the strain CECT5344 are present a wide variety of gene clusters putatively involved in resistance to metals and drugs [23,24]. Most of these metal resistance genes are involved in copper extrusion, but also there are efflux pumps for other metals like zinc or nickel and even for drugs and organic compound like antibiotics or acriflavin. *P*. *pseudoalcaligenes* CECT5344 contains at least 18 gene clusters putatively involved in metal resistance, including 10 clusters belonging to the proton antiporter RND family, two clusters of the chemiosmotic gradient H^+^/K^+^-dependent cation diffusion facilitator (CDF) family, four clusters encoding efflux P1-type ATPases, and two clusters that do not show homology with metal extrusion transporters. The RND efflux pumps are composed of an outer membrane protein, a periplasmic component with a small membrane hydrophobic region and an integral membrane component. They can be classified into two subgroups accordingly to several conserved residues: the RND-HME subclass is clearly involved in extrusion of metals like copper, zinc, cobalt, nickel and silver, whereas the RND-HAE subclass is related with extrusion of antibiotics and aromatic compounds [16]. Among the 10 RND efflux transporters of *P*. *pseudoalcaligenes* CECT5344, five belong to the RND-HME subclass for divalent cations extrusion and five correspond to the RND-HAE subclass for antibiotics efflux [23,24]. In the proteomic analysis by LC-MS/MS carried out in this work, several efflux pumps belonging to P1-type ATPases, RND-HME and RND-HAE systems for metal extrusion have been found induced by the cyanide-containing wastewater from the jewelry industry (Fig 1, Table 2), suggesting that the strain CECT5344 displays a great variety of metal efflux pumps to deal with high metal concentrations that may be active in the presence of this industrial residue. Additionally, the ferric-uptake system FhuC (ATP-binding component of a hydroxamate-type siderophore import system) was also induced by the industrial residue (Fig 3E and S2 Fig, Table 2). Siderophores are secreted when iron availability is scarce inside the cells, and the Fe^3+^-siderophores are transported inside the cells by hydroxamate-or catechol-type siderophore transporters [4].

Glucans biosynthesis is an important process for the correct cell-wall and membrane functionality, and therefore for cell viability [43]. Undecaprenyl-diphosphate is a precursor in the peptidoglycan biosynthesis, and it has been reported in *Cupriavidus metallidurans* CH34 that the undecaprenyl-diphosphate phosphatase PbrB cooperates with a Zn/Cd/Pb efflux pump P-type ATPase to allow bacterial lead resistance [44]. In this context, the induction of two glucan biosynthesis proteins by the jewelry residue in *P*. *pseudoalcaligenes* CECT5344 (Fig 2F) may be also related with the metal resistance mechanisms operating in this bacterium. Additionally, proteins related with polyhydroxyalkanoate (PHA) metabolism like the granule-associated protein PhaF (phasin) and the class II PHA synthase PhaC1 have been found induced by the cyanide-containing residue (Fig 2E). Synthesis of PHA as by-product during industrial cyanide removal is an added value to the bioremediation process [45].

It is remarkable that a relatively high number of genes coding for gluconate-operon repressor (GntR) family transcriptional regulators, which are phylogenetically related (S3 Fig), are located in the *P*. *pseudoalcaligenes* CECT5344 chromosomal regions that contains genes induced by the jewelry residue. GntR regulators contain a highly conserved N-terminal helix-turn-helix (HTH) domain for DNA binding and a variable effector binding/oligomerization C-terminal domain that provides the basis for their classification into the AraR, DevA, FadR, HutC, MocR, PlmA and YtrA subfamilies [46,47]. The effector molecule binds to the C-terminal domain causing a conformational change that affect GntR binding to the palindromic DNA sequence motifs present in target genes. GntR proteins control multiple biological processes in different bacterial species, including virulence [48], oxidative stress and iron uptake [49], resistance to quinolones and β-lactams [50], and γ-ray and UV radiation resistance [51]. A GntR family regulator also controls mycobacterial drug resistance acting as a copper-responsive global repressor [52]. In *P*. *pseudoalcaligenes* CECT5344, some genes coding for GntR regulatory proteins are clustered together structural genes for different oxidases and metal transport systems, suggesting that this type of regulators may play a role controlling the processes required for cyanide tolerance/assimilation and metal resistance. The phenotype of a *P*. *pseudoalcaligenes* CECT5344 GntR^─^ mutant (Fig 4) is consistent with a plausible role of the GntR regulators in controlling cyanide assimilation, although this finding deserves future investigations.

The proteomic study by LC-MS/MS carried out in this work has corroborated the transcriptomic data previously available [25]. Thus, more than 90% of the proteins induced by the jewelry residue (when compared to ammonium) are encoded by genes that were found induced in the DNA microarrays in response to this industrial residue, providing evidences that transcribed genes are also translated to protein (Fig 1). In addition, the proteomic analysis has been very useful detecting proteins that are exclusively present in the cyanide-containing wastewater and may play a relevant role in metal and cyanide resistance and detoxification (Table 2).

In addition, in this work it has been demonstrated that the strain CECT5344 was able to detoxify under alkaline conditions (pH 9.0) a liquid residue from the jewelry industry that contains a high concentration of cyanide (12 mM) and metals (Fig 5). In a previous work, degradation of only 2 mM sodium cyanide was carried out in a batch reactor by the strain CECT5344 under alkaline conditions [21]. Bacterial consortia able to use metal-bound cyanides as nitrogen source, including copper and zinc complexes has been described and used in detoxification of cyanide from electroplating wastewater [5,6]. Several *Pseudomonas* species isolated from a copper mine have been also reported to degrade efficiently cyanide [53]. Detoxification of cyanide from industrial effluents is not restricted to bacterial strains, and fungi or algae like *Scenedesmus obliquus* have been used with this purpose [54,55]. Many of the microorganisms used in bio-treatments of effluents contaminated with cyanide share the requirement of oxygen, but a few other processes for cyanide removal operate under anaerobic conditions [56]. However, most biological treatments described so far are based on microorganisms that work at neutral pH [7,8,9,57,58]. By contrast, we have shown in this work that the alkaliphilic bacterium *P*. *pseudoalcaligenes* CECT5344 is able to remove free and metal−cyanide complexes from jewelry industry wastewaters in a batch reactor operating at alkaline conditions (pH 9.0), which was kept constant during the degradation process (Fig 5). An alkaline pH to prevent HCN volatilization is a key factor for cyanide degradation in bioreactors and must be considered to increase efficiency and safety of the process. The addition of citrate to the culture media has been demonstrated to be required for the removal of high concentration of cyanide by the strain CECT5344 (Fig 5). This tricarboxylic acid may act as metal chelator contributing to reduce metal toxicity for this bacterium. In this context, citrate is a well-known metal chelator [59] and it has been also described as a bacterial siderophore [60]. Furthermore, citrate and other siderophores/metallophores not only may transport metals, but also may play a variety of other functions as toxic metal sequestration, protection from oxidative stress and molecular signalling [61].

In summary, the proteomic approach by quantitative LC-MS/MS has allowed the identification of key metabolic enzymes, regulators and metal transporters in *P*. *pseudoalcaligenes* CECT5344. This work establishes the basic knowledge to a better understanding of the whole process of cyanide degradation and assimilation by the strain CECT5344, and it may be used to improve the bioremediation of cyanide-containing wastewaters. The batch reactor bioremediation of the jewelry liquid residue described in this work may offer an effective alternative to the existing physico-chemical treatments for detoxification of wastewaters containing free cyanide and/or metal–cyanide complexes, considering that the *P*. *pseudoalcaligenes* strain CECT5344 has proved to degrade at alkaline pH, not only free cyanide but also metal–cyanide complexes present in jewelry wastewaters.

## Supporting information

S1 Fig Functional analysis of *Pseudomonas pseudoalcaligenes* CECT5344 proteins induced in a comparative study jewelry residue versus ammonium as nitrogen source. (TIF)

S2 Fig Functional analysis of *Pseudomonas pseudoalcaligenes* CECT5344 proteins exclusively detected in the jewelry residue. (TIF)

S3 Fig Phylogenetic tree of GntR-like regulatory proteins of *Pseudomonas pseudoalcaligenes* CECT5344. (TIF)

S1 Appendix Supporting information figure legends and references. (PDF)
