# Supplementary figures and images for: Quantitative proteomic analysis of Pseudomonas pseudoalcaligenes CECT5344 in response to industrial cyanide-containing wastewaters using Liquid Chromatography-Mass Spectrometry/Mass Spectrometry (LC-MS/MS)
Source: PLoS One. 2017 Mar 2;12(3):e0172908. doi: 10.1371/journal.pone.0172908 (PMC5333837; doi:10.1371/journal.pone.0172908)

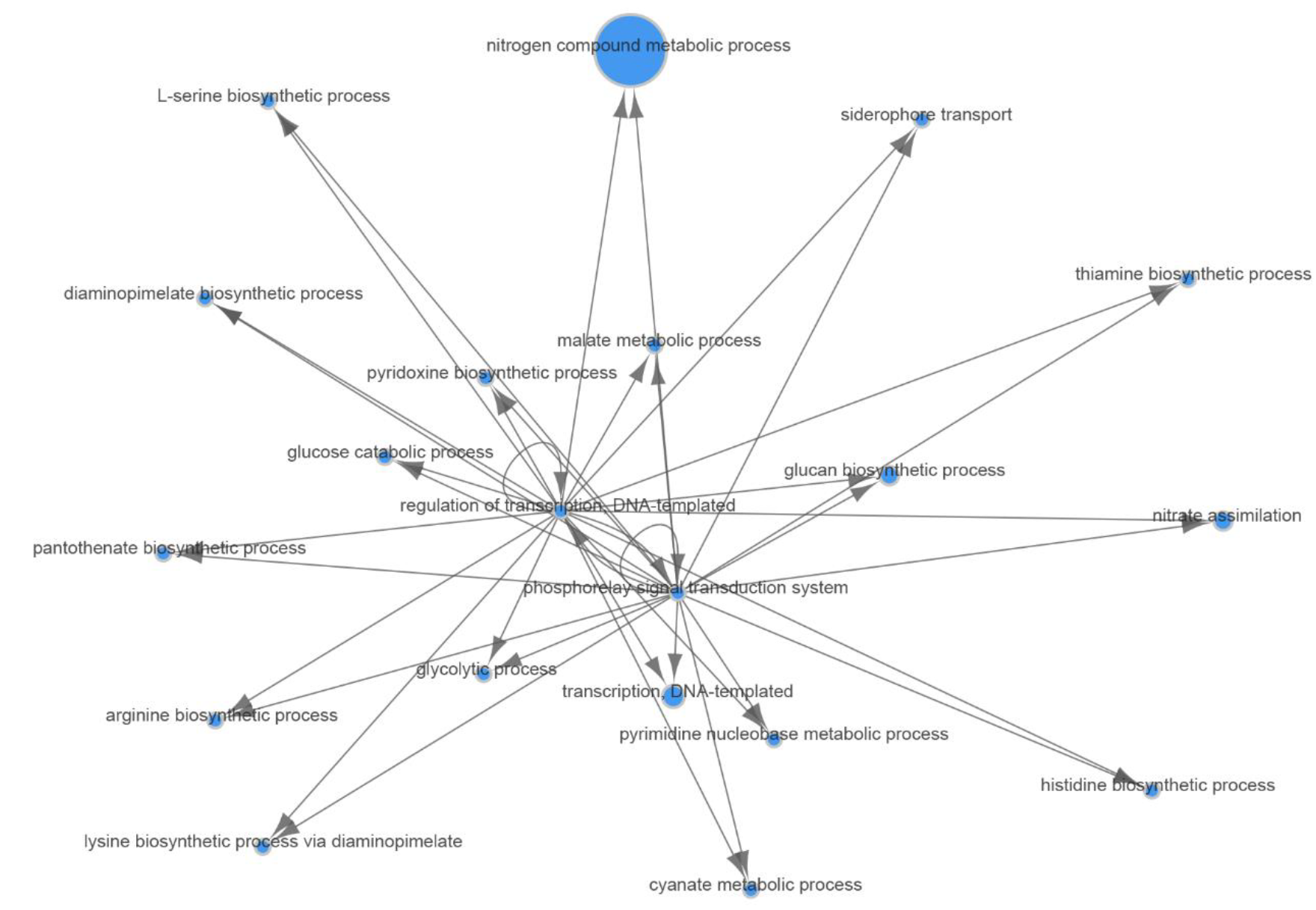

Supplement: S1 Fig — (TIF) [file pone.0172908.s001.tif]

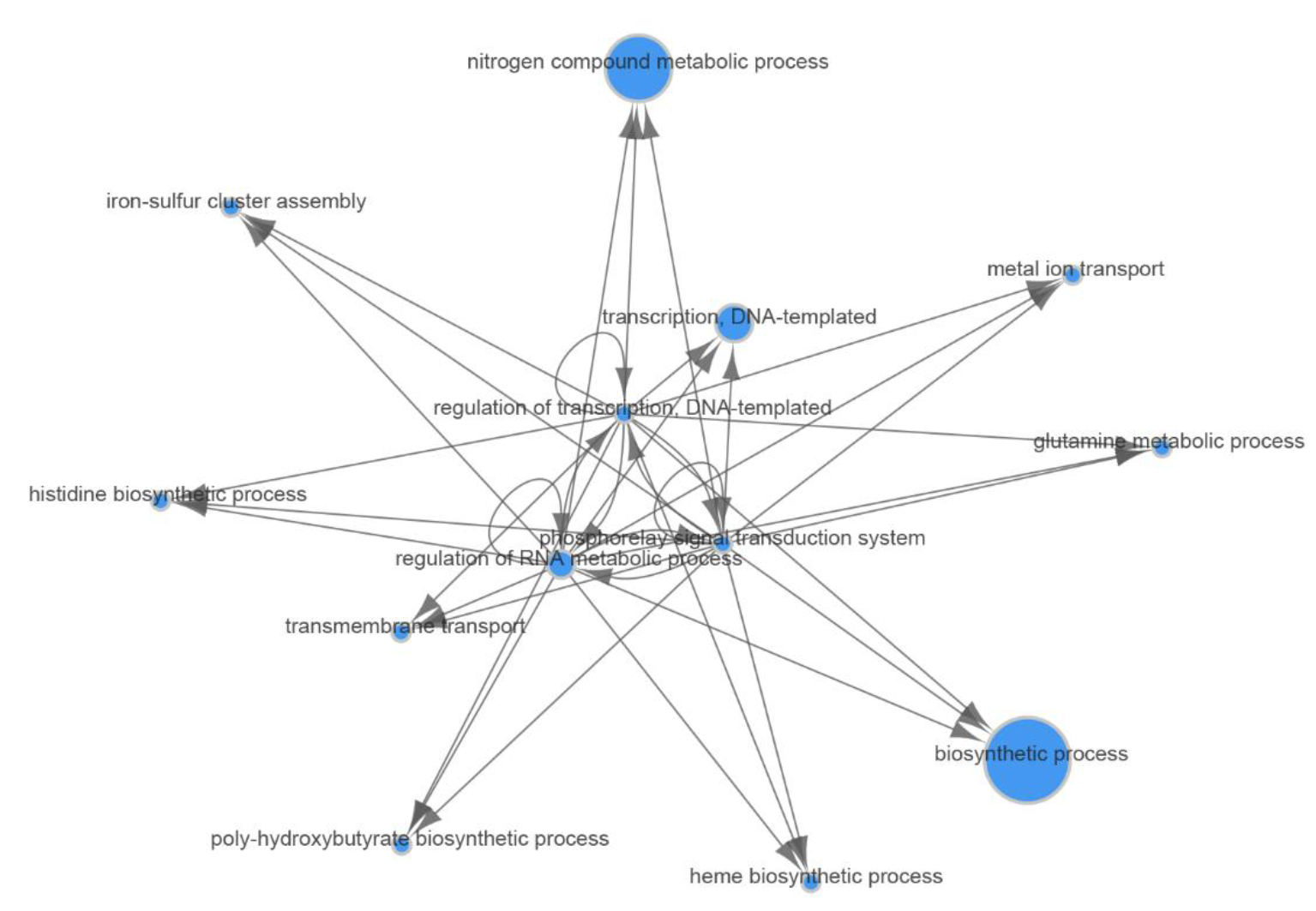

Supplement: S2 Fig — (TIF) [file pone.0172908.s002.tif]

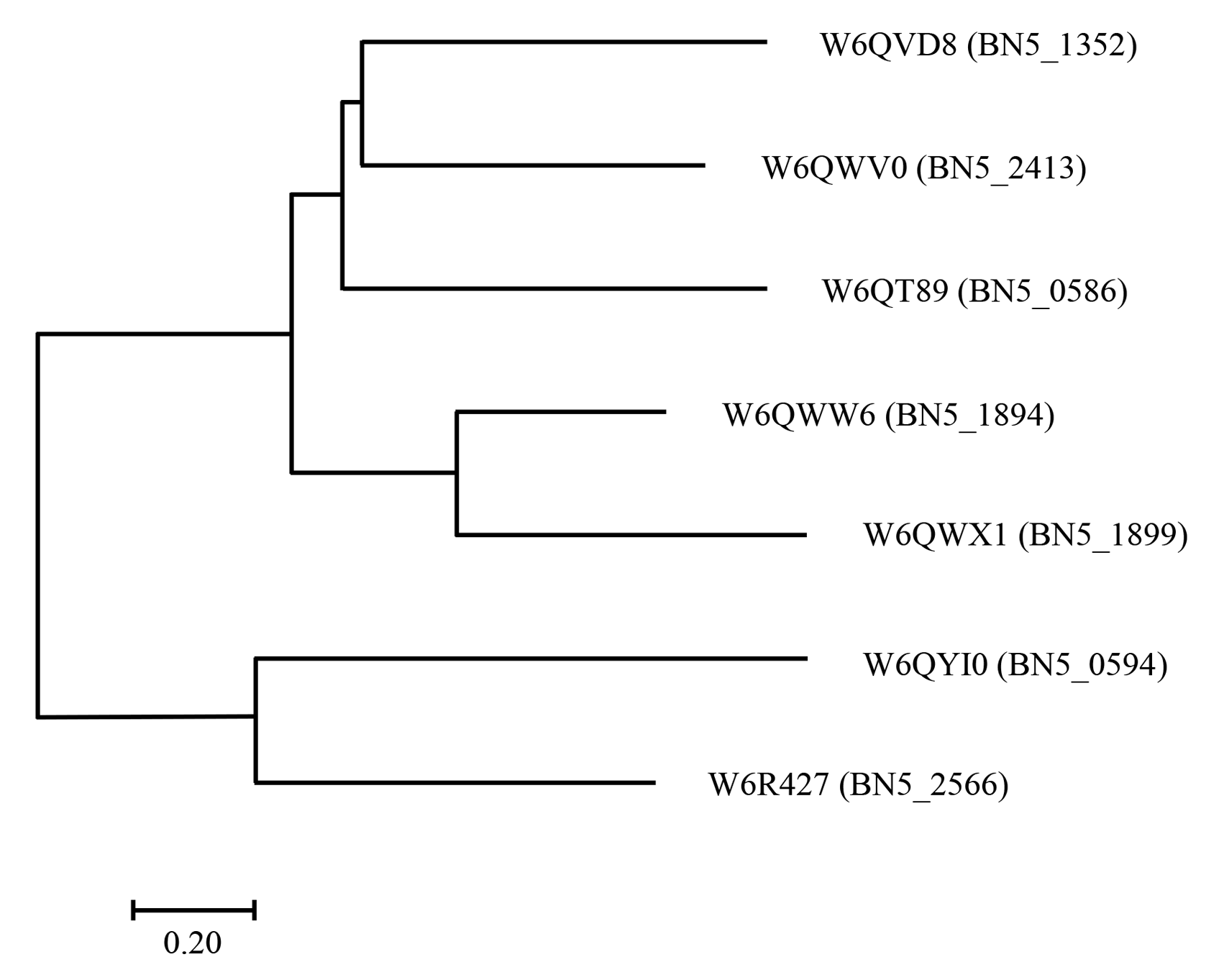

Supplement: S3 Fig — (TIF) [file pone.0172908.s003.tif]
