# Supplementary material for: Quantitative proteomic analysis of Pseudomonas pseudoalcaligenes CECT5344 in response to industrial cyanide-containing wastewaters using Liquid Chromatography-Mass Spectrometry/Mass Spectrometry (LC-MS/MS)
Source: PLoS One. 2017 Mar 2;12(3):e0172908. doi: 10.1371/journal.pone.0172908 (PMC5333837; doi:10.1371/journal.pone.0172908)
Supplement: S1 Appendix — (PDF) [file pone.0172908.s004.pdf]

Supporting Information

Figure Legends

S1 Fig. Functional analysis of *Pseudomonas pseudoalcaligenes* CECT5344 proteins induced in a comparative study jewelry residue versus ammonium as nitrogen source. The GO analysis has been carried out with the Comparative GO software and 20 categories have been established: nitrogen compound metabolic process, transcription/DNA-templated, nitrate assimilation, glucan biosynthetic process, siderophore transport, thiamine biosynthetic process, histidine biosynthetic process, pyrimidine nucleobase metabolic process, cyanate metabolic process, glycolytic process, lysine biosynthetic process via diaminopimelate, arginine biosynthetic process, pantothenate biosynthetic process, glucose catabolic process, pyridoxine biosynthetic process, diaminopimelate biosynthetic process, L-serine biosynthetic process, malate metabolic process, regulation of transcription/DNA-templated and phosphorelay signal transduction system. From all proteins induced by the jewelry residue (when compared to ammonium grown cells), only those displaying a fold change > 2 and *p*-value < 0.05 have been considered. The size of the blue balls represents the number of proteins.

S2 Fig. Functional analysis of *Pseudomonas pseudoalcaligenes* CECT5344 proteins exclusively detected in the jewelry residue. The GO analysis has been carried out with the Comparative GO software and 13 categories have been established: biosynthetic process, nitrogen compound metabolic process, transcription/DNA-templated, nitrate assimilation, regulation of RNA metabolic process, metal ion transport, glutamine metabolic process, regulation of transcription/DNA-template, phosphorelay signal transduction system, heme biosynthesis process, poly-hydroxybutyrate biosynthetic process, transmembrane transport, histidine biosynthetic process and iron-sulfur cluster

assembly. From all proteins exclusively found in the jewelry residue, only those displaying a fold change < 2 (variation within biological replicates) have been considered when. The *p*-value is not provided in this descriptive analysis. The size of the blue balls represents the number of proteins.

S3 Fig. Phylogenetic tree of GntR-like regulatory proteins of *Pseudomonas pseudoalcaligenes* CECT5344. The molecular phylogenetic analysis has been carried out by the Maximum Likehood method. The evolutionary history was inferred by using the Maximum Likelihood method based on the JTT matrix-based model [1]. The tree with the highest log likelihood (-1903.5489) is shown. Initial tree(s) for the heuristic search were obtained automatically by applying Neighbor-Join and BioNJ algorithms to a matrix of pairwise distances estimated using a JTT model, and then selecting the topology with superior log likelihood value. The tree is drawn to scale, with branch lengths measured in the number of substitutions per site. All positions containing gaps and missing data were eliminated. There were a total of 95 positions in the fine dataset. Evolutionary analyses were conducted in MEGA7 [2]. Gene names correspond to the accession number HG916826 [3].

References

1. Jones DT, Taylor WR, Thornton JM (1992) The rapid generation of mutation data matrices from protein sequences. Comput Appl Biosci 8: 275-282.
2. Kumar S, Stecher G, Tamura K (2016) MEGA7: Molecular Evolutionary Genetics Analysis version 7.0 for bigger datasets. Mol Biol Evol 33: 1870-1874.
3. Wibberg D, Luque-Almagro VM, Igeño MI, Bremges A, Roldán MD, et al. (2014) Complete genome sequence of the cyanide-degrading bacterium *Pseudomonas pseudoalcaligenes* CECT5344. J Biotechnol 175: 67-68.
